# COVID‐19 Vaccine Effectiveness Against Hospitalizations and Severe Outcomes in Kosovo, 2022–2024: A Test‐Negative Case–Control Study

**DOI:** 10.1111/irv.70152

**Published:** 2025-09-08

**Authors:** Besfort Kryeziu, Sandra Cohuet, Ariana Kalaveshi‐Osmani, Zana Kaçaniku‐Deva, Pranvera Kaçaniku‐Gunga, Iris Finci, Miguel Angel Sanchez, James Humphreys, Naser Ramadani, Edita Haxhiu, Kostas Danis, Angela M. C. Rose, Isme Humolli, Mark A. Katz

**Affiliations:** ^1^ National Institute of Public Health of Kosovo Prishtina Kosovo; ^2^ The Mediterranean and Black Sea Programme for Intervention Epidemiology Training (MediPIET) European Centre for Disease Prevention and Control (ECDC) Stockholm Sweden; ^3^ University “Hasan Prishtina” Faculty of Medicine Pristina Kosovo; ^4^ Epiconcept Paris France; ^5^ World Health Organization Regional Office for Europe Copenhagen Denmark; ^6^ World Health Organization Liaison Office Kosovo Pristina Kosovo

**Keywords:** COVID‐19, hospitalization, Kosovo, severe outcomes, vaccine effectiveness

## Abstract

**Background:**

Few studies have evaluated COVID‐19 vaccine effectiveness (VE) in middle‐income countries, particularly in eastern Europe. We aimed to estimate COVID‐19 VE against SARS‐CoV‐2‐confirmed hospitalizations and severe outcomes in Kosovo.

**Methods:**

We conducted a test‐negative case–control study using data from Kosovo's severe acute respiratory infection (SARI) sentinel surveillance system from January 2022 to June 2024. We enrolled adult patients aged ≥ 18 years hospitalized with SARI. From all patients, we collected clinical data, vaccination history, and a nasopharyngeal specimen, which was tested for SARS‐CoV‐2 using RT‐PCR. SARS‐CoV‐2‐positive patients were cases; those testing negative were controls. We estimated VE overall and against severe outcomes (requiring oxygen, intensive care admission, or in‐hospital death) using logistic regression, adjusting for age, sex, and comorbidities, calculating VE as (1–adjusted odds ratio) × 100.

**Results:**

We included 564 SARI patients; 218 (39%) tested positive for SARS‐CoV‐2. Overall, 24% of SARI patients had received at least one COVID‐19 vaccine dose in the previous 12 months. VE against SARS‐CoV‐2‐confirmed SARI hospitalization among all adults was 72% (95% CI: 30%–89%) at 14–179‐day postvaccination, and 26% (95% CI: −33%–59%) at 180–364 days. In adults ≥ 60 years, VE was 52% (95% CI:−31%–82%) at 14–179‐day postvaccination, and −36% (95% CI: −190%–36%) at 180–364 days. VE against severe outcomes was 67% (95% CI: −14%–91%) at 14–179 days, and 17% (95% CI:−111%–67%) at 180–364 days.

**Conclusions:**

Our findings suggest that COVID‐19 vaccination in Kosovo offered substantial protection against hospitalization and severe outcomes within 6 months, though confidence intervals were wide for some subgroups. Effectiveness waned after 6 months, highlighting the need for periodic booster doses.

## 1 Introduction

Since the beginning of the pandemic in 2020, COVID‐19 has caused over 7 million deaths and over 771 million infections worldwide. Since the COVID‐19 Public Health Emergency of International Concern was declared in May 2023, SARS‐CoV‐2 has continued to cause hospitalizations and deaths [1, 2, 3].

COVID‐19 vaccination is estimated to have saved 19.8 million lives globally and 1.4 million lives in Europe alone during the pandemic [4]; COVID‐19 vaccination continues to be effective in preventing severe disease and death [5].

Kosovo [6] has a population of 1.5 million people and has an upper middle‐income economy. Kosovo's first vaccine against COVID‐19 was administered on March 29, 2021 [7]. Since then, over 1.8 million vaccine doses have been administered. In the COVID‐19 vaccination campaign in Kosovo, BNT162b2 (Pfizer‐BioNTech) vaccine was the most widely used vaccine, followed by ChAdOx1 nCoV‐19 (Oxford‐AstraZeneca), which was mainly available in the early months of the vaccination campaign. COVID‐19 vaccination was initially targeted at healthcare workers, individuals aged > 65 years, and those with comorbidities. The campaign was later expanded to include all adults aged 18 years and older, and subsequently, adolescents aged ≥ 12 years old. Children 5–11 years (with parental consent) were added in 2022 (Table S1). Overall, 46% of the population received primary series vaccination, 6% received at least one booster, and 4% received two boosters [8]. However, few vaccines were administered during 2023 and 2024 [9].

Estimating COVID‐19 vaccine effectiveness (VE) on an ongoing basis, particularly with local data, is critical to guide COVID‐19 vaccine policies. As of early 2025, most COVID‐19 VE studies had been conducted in high‐income countries, where COVID‐19 vaccination coverage was much higher than in low‐ and middle‐income countries (LMICs) [10, 11]. In LMICs, where population characteristics differ, and population immunity and circulating SARS‐CoV‐2 strains may vary, COVID‐19 VE may also differ [12]. As of March 2025, only one analysis of COVID‐19 VE from Kosovo had been published, which evaluated VE during a 3‐month period in 2021 [13], and few COVID‐19 VE studies had been published in other middle‐income countries in the eastern part of the WHO European region [14].

We used data from our enhanced SARI surveillance system to estimate COVID‐19 VE against hospitalizations for severe acute respiratory infections (SARI) and against more severe outcomes, which we defined as requiring oxygen, mechanical ventilation, intensive care unit (ICU) admission, and/or death.

## 2 Methods

### 2.1 Study Design

We conducted a case–control study using the test‐negative design methodology. We designed our study following the approach outlined in the WHO/Europe guidance document on studies to evaluate vaccine effectiveness against SARI [15].

### 2.2 Study Population

The study population consisted of adults (≥ 18 years old) who were admitted to any of the four SARI sentinel surveillance hospitals in Kosovo from January 1, 2022 to June 30, 2024 and who met the WHO case definition for SARI, defined as an acute respiratory infection with a history of fever or measured fever of ≥ 38°C, and cough, with onset within the past 10 days, that requires hospitalization [16]. Of those sentinel surveillance hospitals, three were located in Prishtina (The Infectious Diseases Clinic, the Pulmonology Clinic, and the Central Intensive Care Medicine) and one in Prizren (Prizren Regional Hospital). Together, these four hospitals cover 48% of the population in Kosovo [17].

Patients were excluded from the study if they had incomplete or missing data on vaccination dates or status, were vaccinated < 1 week before symptom onset, or had discrepant or missing data regarding the date of swab collection, symptom onset, or hospitalization. We also excluded healthcare workers out of concerns that they had increased exposure to SARS‐CoV‐2 viruses compared with the general population.

### 2.3 Data Collection and Recruitment Algorithms

For every SARI patient, we administered a structured questionnaire directly to the patient, which included questions about the patient's demographics and medical history, history of acute illness, and history of COVID‐19 and influenza vaccinations. In addition, the study staff obtained information about the patient's clinical course in the hospital, and whether the patient was discharged home, transferred to another hospital, or died in the hospital, by reviewing the patient's medical chart.

We collected a nasopharyngeal or a nasal swab from every patient within 48 h of hospital admission, which was tested by RT‐PCR for SARS‐CoV‐2, influenza, and RSV at the National Microbiology Laboratory in the National Institute of Public Health of Kosovo (NIPHK). SARS‐CoV‐2‐positive specimens with CT (cycle threshold) values < 30 were sent to Eurofins Genomics Europe Pharma and Diagnostics Products and Services Sanger/PCR GmbH in Konstanz, Germany, for whole genomic sequencing.

Study staff verified patients' COVID‐19 vaccination status by cross‐referencing the national vaccination database. All data were stored and managed using Research Electronic Data Capture (REDCap) [18, 19].

### 2.4 Definitions

The primary outcome was COVID‐19 VE against PCR‐confirmed SARS‐CoV‐2 SARI hospitalized patients. We defined cases as SARI patients who were PCR‐positive for SARS‐CoV‐2 and controls as SARI patients who were negative for SARS‐CoV‐2. As a secondary outcome, we estimated annual VE against severe disease. Patients were classified as having “severe disease” if they required oxygen or mechanical ventilation, were admitted to the ICU, or died during hospitalization. For our primary and secondary analyses, the exposure was at least one COVID‐19 vaccine received within the previous 12 months (“annual” VE), but not 0–13 days before symptom onset, regardless of the number of doses. The comparison group was SARI patients who had never been vaccinated or who had not received any COVID‐19 vaccines in the 12 months before their hospital admission for SARI.

We defined prior SARS‐CoV‐2 infection as a positive RT‐PCR test result for SARS‐CoV‐2 ≥ 90 days prior to the current illness. For this variable, we cross‐checked the previous test results of all patients with the national database of SARS‐CoV‐2 PCR tests performed before the study period.

### 2.5 Data Analysis

We conducted a descriptive analysis for the entire study population, including reasons for exclusion, demographic characteristics, number of COVID‐19 doses received, and SARS‐CoV‐2 status. We used the two‐sample test to compare proportions of categorical variables (e.g., age group) among cases and controls, and quantile regression at the median to compare the number of days from last dose to symptom onset, onset to admission, and length of hospital stay among cases and controls.

We calculated crude odds ratios (ORs) and adjusted ORs using multivariable logistic regression. We adjusted the multivariable model for the a priori variables sex (as a categorical variable), onset date (either as onset month or restricted cubic spline of onset date) and age (age group as a categorical variable, or age as either a linear term, or as a restricted cubic spline) and the presence of at least one of 11 chronic medical conditions (diabetes, heart disease, chronic respiratory disease, asthma, cancer, immunodepression, rheumatic disorders, liver disease, renal disease, anemia, and tuberculosis). We also adjusted for the following potential confounders: prior COVID‐19 infection, smoking, pregnancy, and obesity (body mass index (BMI) ≥ 30, calculated from measured or reported height and weight of patients). We selected the model with the best functional form, based on the Akaike Information Criterion. We calculated VE using the following formula: VE = (1—OR)*100. A 95% confidence interval (CI) was calculated for each estimate.

We estimated VE for the last vaccine received 14–179 days prior to symptom onset and for the last vaccine received 180–364 days prior to symptom onset. Individuals vaccinated 0–13 days prior to symptom onset were excluded from the analysis to avoid misclassification during the interval before the anticipated onset of immune protection of the vaccine. We estimated VE (i) in all adults ≥ 18 years old; (ii) in adults ≥ 60 years old; and (iii) against severe outcomes in all adults ≥ 18 years old. We described circulating Omicron subvariants using publicly accessible data from the Global Initiative on Sharing All Influenza Data (GISAID) [20]. We performed the analysis using R (version 4.1.3).

### 2.6 Ethical Considerations

Data collection for this VE study was conducted within the existing SARI surveillance, which constitutes routine public health practice regulated by Law 08/L‐200 on Prevention and Control of Infectious Diseases. Ethical committee approvals were obtained from (i) the Ethics Committee of the National Institute of Public Health of Kosovo (Protocol ID 51/2021), (ii) the Ethics Committee of the Chamber of Doctors of Kosovo (Protocol ID 57/2021), and (iii) the WHO Research Ethics Review Committee (Protocol ID CERC.0098B).

## 3 Results

### 3.1 Study Population

**FIGURE 1:**
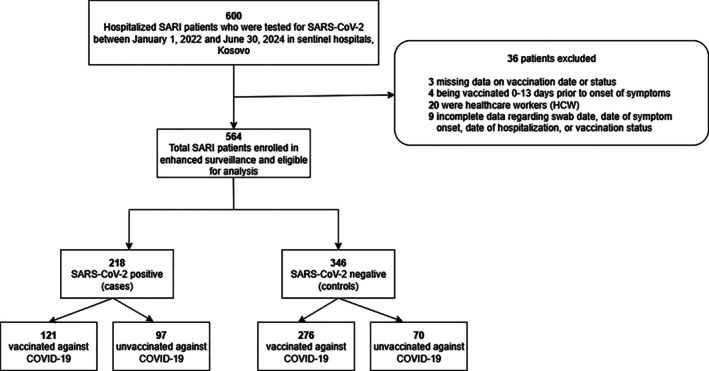
Flowchart of patient recruitment and exclusion for COVID‐19 vaccine effectiveness analysis in Kosovo, January 1, 2022–June 30, 2024.

Between January 1, 2022 and June 30, 2024, 600 SARI patients were enrolled in the study. Overall, 36 patients were excluded due to (i) missing data on vaccination date or status (*n* = 3), (ii) being vaccinated 0–13 days prior to onset of symptoms (*n* = 4), (iii) being healthcare workers (*n* = 20), and/or (iv) incomplete data regarding swab date, date of symptom onset, date of hospitalization, or vaccination status (*n* = 9) (Figure 1). In the final analysis, we included 564 SARI patients.

The median age of all participants was 63 years (Interquartile Range [IQR]: 46–74); [288 (51%)] SARI patients were female, and 282 (50%) had at least one chronic disease (Table 1). Overall, 136 (24%) SARI patients had received at least one COVID‐19 vaccine in the past 12 months. Among the 136 SARI patients who had received a COVID‐19 vaccine dose in the previous 12 months, 89 (66%) vaccines were primary vaccine series, 41 (30%) were first booster doses, and 6 (4%) were second booster doses. Of all vaccinated SARI patients, 20 (4%) had received only one COVID‐19 vaccine, 377 (67%) had received two doses, and 94 (17%) had received three doses. In total, 87 (40%) cases had received the complete primary series compared with 196 (57%) controls. Of the primary vaccine series received, 241 (85%) were BNT162b2, while 40 (14%) were ChAdOx1‐nCoV‐19; all booster doses were BNT162b2 (Table 1). Of all SARI patients, 33 (6%) had been vaccinated against influenza in the previous and current influenza seasons.

**TABLE 1:** Demographic and clinical characteristics of SARI patients enrolled in COVID‐19 vaccine effectiveness study in Kosovo, January 1, 2022–June 30, 2024.

Variables		Overall study sample (*N* = 564)		Number of laboratory‐confirmed SARS‐CoV‐2 cases (*N* = 218)		Number of test‐negative SARS‐CoV‐2 controls (*N* = 346)		*p*‐Value
		*n*	%	*n*	%	*n*	%	
Median age (years), (IQR)		63 (46–74)		65 (52–75)		61 (43–72)		0.0189
Age group	18–49	162	29	50	23	112	32	0.2446
	50–59	75	13	26	12	49	14	0.8082
	60–69	120	21	49	22	71	21	0.8956
	70–79	145	26	66	30	79	23	0.3397
	≥ 80	62	11	27	12	35	10	0.8019
Sex	Male	276	49	112	51	164	47	0.5138
	Female	288	51	106	49	182	53	
	Pregnant	8	3	3	3	5	3	
Chronic conditions*	None	282	50	110	50	172	50	1.000
	At least one	282	50	108	50	174	50	
	Cancer	30	5	13	6	17	5	0.9047
	Hypertension	276	49	111	51	165	48	0.6250
	Other cardiovascular	85	15	29	13	56	16	0.7133
	Diabetes	158	28	57	26	101	29	0.6864
	Liver	11	2	7	3	4	1	0.8305
	Lung disease and asthma	94	17	39	18	55	16	0.7984
	Neurological	12	2	6	3	6	2	0.9117
	Others^1^	181	32	74	34	107	31	0.6711
Smoking	Yes, current smoker	115	20	40	18	75	22	0.6135
	Previous smoker (quit ≥ 1 year ago)	127	23	55	25	72	21	0.5941
Previous SARS‐CoV‐2 infection	No	559	99	216	99	343	99	1.000
	Yes	3	1	1	0	2	1	
	(Missing information)	2	0	1	0	1	0	
At least one dose in the previous 12 months		136	24	65	30	71	20	
Received influenza vaccination in previous season	No	531	94	206	94	325	94	1.000
	Yes	33	6	12	6	21	6	
COVID‐19 vaccination status	Unvaccinated	167	30	97	44	70	20	
	Partial primary series (one dose only)	20	4	12	6	8	2	0.6692
	Complete primary series (two doses)	283	50	87	40	196	57	0.0083
	Complete primary series + first booster (three doses)	85	15	18	8	67	19	0.2663
	Complete primary series + first and second booster (four doses)	9	1	4	2	5	1	0.9006
Vaccine types (by number of doses)								
First dose	Moderna	2	1	1	1	1	0	
	ChAdOx1‐nCoV‐19	67	17	22	18	45	16	0.8365
	BNT162b2	328	83	98	81	230	83	0.6633
Second dose	Moderna	2	1	1	1	1	0	
	ChAdOx1‐nCoV‐19	64	17	21	19	43	16	0.7641
	BNT162b2	311	82	87	80	224	84	0.4006
First booster (third dose)	ChAdOx1‐nCoV‐19	4	4	0	0	4	6	
	BNT162b2	90	96	22	100	68	94	0.2396
Second booster (fourth dose)	BNT162b2	9	100	4	100	5	100	
Median days since last dose to symptom onset (IQR)		521 (292–734)		341 (220–514)		578 (363–799)		< 0.0001
Clinical outcome	Days between symptom onset and hospital admission (median [IQR])	6 [4–8]		6 (4–7)		6 (5–8)		1.000
	Median length of hospital stays in days (IQR)	9 [6–14]		9 [6–14]		9 (6–14)		1.000
	Admitted to ICU	8	1	3	1	5	1	1.000
	Respiratory support^2^	244	43	105	48	139	40	0.2119
	In‐hospital death	40	7	24	11	16	5	0.5073

^1^ Others include obesity (BMI ≥ 30), renal diseases, immunodeficiencies, rheumatological diseases, hematological diseases, and thyroid‐related health issues.

^2^ Respiratory support includes oxygen administration, CPAP, and mechanical ventilation.

* One patient may have multiple chronic medical conditions, so the total percentages may add up to more than 100%.

BMI, body mass index; CPAP, continued positive airway pressure; ICU, intensive care unit; IQR, interquartile range; SARI, severe acute respiratory infection.

Overall, 218 (39%) patients were SARS‐CoV‐2 positive (cases), and 346 (61%) were SARS‐CoV‐2 negative (controls). In total, 59 (12%) patients were positive for influenza, and 5 (1%) were positive for RSV. In all, 244 (43%) SARI patients had a severe outcome, including 40 (7%) who died (Table 1).

Controls were slightly younger (median age: 61 years old [IQR: 43–72]) than cases (median age: 65 years old [IQR: 52–75]). There was a slightly higher proportion of males (51%) among cases compared with controls (47%). The percentage of cases and controls with at least one chronic condition was similar.

The number of days between symptom onset and hospital admission was 6 days for cases and controls. The median length of hospital stay was 9 days [IQR: 6–14] for cases and 8 days [IQR: 6–14] for controls. In total, 105 (48%) cases received respiratory support (oxygen administration and/or mechanical ventilation) compared to 139 (40%) of controls, and 24 (11%) cases had a fatal outcome compared to 16 (5%) controls (Table 1).

**FIGURE 2:**
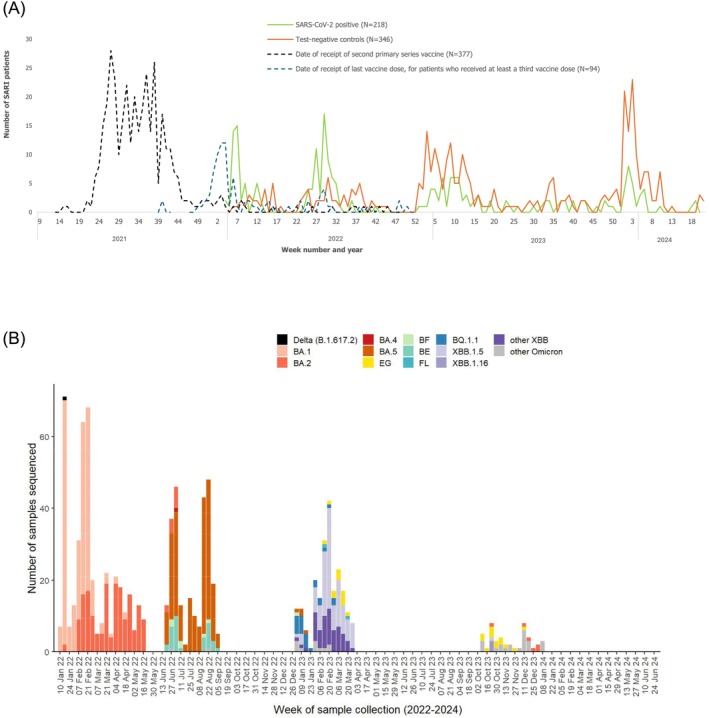
(a) Year and week of sample collection of SARS‐CoV‐2‐positive cases and SARS‐CoV‐2‐negative controls, and dates of receipt of second primary series vaccine and last booster vaccine dose among vaccinated SARI patients included in COVID‐19 vaccine effectiveness study in Kosovo, January 1, 2022–June 30, 2024. (b) Sequencing data from SARS‐CoV‐2‐positive samples in Kosovo during the study analysis period, January 1, 2022–June 30, 2024 (Data downloaded from GISAID on January 31, 2025) * The number of sequenced samples for the given period in Kosovo was *n* = 956, where *n* = 373 were from SARI sentinel surveillance sites.

Most patients were enrolled in early 2022, during the summer months of 2022, in late 2023, and in early 2024 (Figure 2a). Overall, between January 2022 and June 2024, 956 SARS‐CoV‐2–positive samples collected in Kosovo, including 373 samples from SARI sentinel surveillance sites, were sequenced. Omicron subvariants predominated throughout the study period. The most frequently detected sublineages were BA.2 and BA.5 in 2022, followed by XBB and JN.1 lineages in 2023–2024 (Figure 2b).

### 3.2 Vaccine Effectiveness

VE against SARS‐CoV‐2‐confirmed hospitalization for SARI among adults ≥ 18 years was 72% (95% CI 30%–89%) at 14–179 days after the receipt of the last COVID‐19 vaccine, 26% (95% CI −33%–59%) at 180–364 days, and 45% (95% CI 6%–68%) for the whole period (14–365 days). Among adults ≥ 60 years, VE was 52% (95% CI −13%–82%) at 14–179 days, −36% (95% CI −190%–36%) at 180–364 days, and 5% (95% CI −85%–51%) for the whole period (14–365 days). Against severe outcomes, VE among adults ≥ 18 years was 67% (95% CI −14%–91%) at 14–179 days, 17% (95% CI −111%–67%) at 180–364 days, and 42% (95% CI −30%–74%) for the whole period (14–365 days) (Table 2, Table S2 and Figure S1).

**TABLE 2:** Annual COVID‐19 vaccine effectiveness against (A) SARS‐CoV‐2‐confirmed hospitalization for SARI among adults ≥ 18 years old, (B) SARS‐CoV‐2‐confirmed hospitalization for SARI among adults ≥ 60 years old, and (C) SARS‐CoV‐2 confirmed severe outcomes among adults ≥ 18 years old, by time since vaccination, Kosovo, January 1, 2022–June 30, 2024.

Days from last vaccine to symptom onset	Cases		Controls		Adjusted VE (95% CI)
	Unvaccinated	Vaccinated	Unvaccinated	Vaccinated	
(A) COVID VE against SARS‐CoV‐2‐confirmed hospitalization for SARI among adults ≥ 18 years					
14–179	153	15	275	28	72% (30%–89%)
180–364	153	49	275	42	26% (−33%–59%)
< 365	153	64	275	70	45% (6%–68%)
(B) COVID‐19 VE against SARS‐CoV‐2‐confirmed hospitalization for SARI among adults ≥ 60 years					
14–179	87	12	147	17	52% (−31%–82%)
180–364	87	43	147	20	−36% (−190%–36%)
< 365	87	55	147	37	5% (−85%–51%)
(C) COVID‐19 VE against SARS‐CoV‐2‐confirmed severe outcomes among adults ≥ 18 years					
14–179	74	8	112	15	67% (−14%–91%)
180–364	74	23	112	17	17% (−111%–67%)
< 365	74	31	112	32	42% (−30%–74%)

## 4 Discussion

In our study of COVID‐19 VE in Kosovo over an almost 3‐year period, we found that a COVID‐19 vaccine received in the previous 6 months prevented nearly three out of four hospitalizations and over two out of three severe outcomes. Our findings, which are the first COVID‐19 VE estimates against hospitalizations and severe outcomes in Kosovo after 2021, and among few VE estimates in middle‐income countries and areas in the WHO European region, underscore the importance of receiving a COVID‐19 vaccine to protect against severe illness. The findings align with guidance from the Kosovo Ministry of Health and WHO, both recommending annual COVID‐19 vaccination for high‐risk groups [21, 22].

Our study found that VE against SARS‐CoV‐2‐confirmed hospitalization among SARI patients aged ≥ 18 years varied based on the interval since the most recent vaccine dose. Within 6 months after vaccination, VE remained high, providing robust protection. However, we found a notable decline in effectiveness between 6 months and 1 year following vaccination. Our findings of high early VE followed by waning effectiveness are similar to those reported in a number of published studies from other parts of the WHO European region during periods of Omicron circulation; a study of pooled data from six countries and areas in the European SARI VE (EuroSAVE) Network conducted during 2022–2023, which included data from Kosovo, found that COVID‐19 VE against SARI hospitalization was 60.1% for the last vaccine received 14–89 days before symptom onset, and 60.0% at 90–179 days, but decreased to 7.0% at 180–269 days [14]. A study from the IVY Network showed that the updated 2023–2024 COVID‐19 vaccine conferred 43% effectiveness against COVID‐19‐associated hospitalization among immunocompetent adults aged ≥ 18 years, and 48% among those aged ≥ 65 years, during a period marked by circulation of the Omicron variant. Another study from the European region found that COVID‐19 vaccines had an overall effectiveness of 43% against hospitalization for laboratory‐confirmed SARS‐CoV‐2 for individuals vaccinated within a completed primary series vaccination with the last dose received ≤ 150 days before onset, but VE improved to 59% after a booster dose [23]. A study from the United States found that the mRNA bivalent vaccine had an effectiveness of 71% against hospitalization for SARS‐CoV‐2 of the BA.4/BA.5 sublineage and 49% against XBB for the first 4 months after vaccination, and protection waned thereafter [24]. An observational matched cohort study in England reported that COVID‐19 booster VE against severe COVID‐19‐related outcomes peaked during the first 3 months following the booster dose [25].

Since the WHO declared the end of the public health emergency of international concern (PHEIC) in May 2023, COVID‐19 vaccination rates have persistently been low globally [26, 27]. In our study, only 24% of SARI patients had received a COVID‐19 vaccine in the past 12 months, despite WHO SAGE and Kosovo Ministry of Health recommendations for annual booster doses [28, 29]. Our findings, which indicate both the continued burden of COVID‐19 in Kosovo and the effectiveness of recent COVID‐19 vaccines, underscore the continued importance of COVID‐19 revaccination, particularly for high‐risk individuals; over half of the SARI patients included in our analysis were at high risk—nearly 55% were > 60 years old, and half had at least one chronic disease. Individuals who are at higher risk for poor outcomes from SARS‐CoV‐2 infection should be identified and prioritized for vaccination. Efforts should be made to better understand why more adults in Kosovo, particularly high‐risk individuals, like most SARI patients in our study, are not getting revaccinated according to WHO and Kosovo Ministry of Health guidelines.

Our study had several strengths. We used the existing SARI surveillance system, which employed a standard SARI case definition, and systematic laboratory testing ensured accurate identification of COVID‐19‐related hospitalizations. In addition, we used reliable COVID‐19 vaccination data from a national electronic COVID‐19 vaccine registry, which the Ministry of Health of Kosovo developed at the outset of the COVID‐19 vaccination campaign in 2021.

Our study also had certain limitations. First, test‐negative case–control studies are observational in nature, and as such, the results are subject to potential bias and confounding factors; we adjusted for many of these in the estimation of VE, but there may have been residual confounding from factors for which we did not control. Second, our relatively small sample size resulted in relatively wide confidence intervals in our VE estimates for older adults and for severe outcomes, and therefore these VE estimates should be interpreted with caution. Third, our study measured VE over a period of 2.5 years against a number of different Omicron subvariants; we were not able to estimate VE over more limited periods, against specific subvariants, or for specific vaccine brands because of our relatively small sample size. In addition, because of sample size limitations, we were not able to evaluate VE for periods of less than 6 months following receipt of the last COVID‐19 vaccine. Finally, due to the absence of a fully digitized health information system for surveillance in Kosovo, we had to rely on paper‐based questionnaires, which may have introduced errors. However, we performed periodic data validation and data cleaning, which increased our data completeness and reliability.

## 5 Conclusions and Recommendations

Our findings suggest that the COVID‐19 vaccine in Kosovo offered substantial protection against both hospitalization and severe outcomes during the 6 months after the most recent vaccine dose; though confidence intervals were wide for some subgroups. Our findings indicated that effectiveness waned after 6 months, highlighting the need for periodic revaccination to maintain protection against COVID‐19 against hospitalization and more severe disease, particularly among high‐risk individuals.

## Author Contributions

**Besfort Kryeziu:** conceptualization, methodology, writing – original draft, writing – review and editing, project administration, formal analysis, software, data curation, supervision, investigation, validation, visualization, funding acquisition, resources. **Sandra Cohuet:** writing – review and editing, formal analysis, visualization, software, data curation, validation, methodology. **Ariana Kalaveshi‐Osmani:** writing – review and editing, project administration, funding acquisition, validation. **Zana Kaçaniku‐Deva:** writing – review and editing, resources, validation. **Pranvera Kaçaniku‐Gunga:** writing – review and editing, project administration, resources, validation. **Iris Finci:** writing – review and editing, methodology, software, validation, project administration. **Miguel Angel Sanchez:** writing – review and editing, methodology, software, validation. **James Humphreys:** writing – review and editing, validation, methodology, software. **Naser Ramadani:** writing – review and editing, validation. **Edita Haxhiu:** writing – review and editing, validation. **Kostas Danis:** writing – review and editing, validation, methodology, supervision. **Angela M. C. Rose:** writing – review and editing, methodology, software. **Isme Humolli:** writing – original draft, writing – review and editing, supervision, project administration, funding acquisition, conceptualization, resources, validation, visualization. **Mark A. Katz:** writing – original draft, writing – review and editing, supervision, project administration, validation, resources, software, formal analysis, conceptualization, visualization.

## Disclosure

The views authors expressed in this publication, who are affiliated with WHO, are those of the authors and do not necessarily reflect the views, policies, or positions of the WHO. For the purposes of this abstract and manuscript, all references to “Kosovo” should be understood as “Kosovo” (in accordance with Security Council Resolution 1244 (1999)). The author is a fellow of the MediPIET program, supported financially by the European Centre for Disease Prevention and Control. The views and opinions expressed herein do not state or reflect those of ECDC. ECDC is not responsible for the data and information collation and analysis and cannot be held liable for conclusions or opinions drawn.

## Conflicts of Interest

The authors declare no conflicts of interest.

## Peer Review

The peer review history for this article is available at https://www.webofscience.com/api/gateway/wos/peer‐review/10.1111/irv.70152.

## Supporting information


**Table S1:** Timeline of COVID‐19 vaccination policy, vaccine products, and eligibility criteria in Kosovo, March 2021–May 2023.
**Table S2:** Adjusted Odds Ratios (aORs) corresponding to COVID‐19 vaccine effectiveness estimates against hospitalization and severe outcomes in Kosovo, January 1, 2022–June 30, 2024.
**Figure S1:** Annual COVID‐19 vaccine effectiveness against SARS‐CoV‐2‐confirmed hospitalization for SARI among adults (A) ≥ 18 years old, (B) ≥ 60 years old, and (C) ≥ 18 years old with severe outcomes, by time since vaccination, COVID‐19 vaccine effectiveness study in Kosovo, January 1, 2022–June 30, 2024.

## Data Availability

The data used in this study are sensitive and cannot be made publicly available without breaching patient confidentiality rules. Therefore, individual participant data and a data dictionary are not available to other researchers but can be made available on request.
