# Supplementary material for: COVID‐19 Vaccine Effectiveness Against Hospitalizations and Severe Outcomes in Kosovo, 2022–2024: A Test‐Negative Case–Control Study
Source: Influenza Other Respir Viruses. 2025 Sep 8;19(9):e70152. doi: 10.1111/irv.70152 (PMC12417103; doi:10.1111/irv.70152)
Supplement: Supplementary file 1 — Table S1: Timeline of COVID‐19 vaccination policy, vaccine products, and eligibility criteria in Kosovo, March 2021–May 2023. Table S2: Adjusted Odds Ratios (aORs) corresponding to COVID‐19 vaccine effectiveness estimates against hospitalization and severe outcomes in Kosovo, January 1, 2022–June 30, 2024. Figure S1: Annual COVID‐19 vaccine effectiveness against SARS‐CoV‐2‐confirmed hospitalization for SARI among adults (A) ≥ 18 years old, (B) ≥ 60 years old, and (C) ≥ 18 years old with severe outcomes, by time since vaccination, COVID‐19 vaccine effectiveness study in Kosovo, January 1, 2022–June 30, 2024. [file IRV-19-e70152-s001.docx]

**Supplementary Table 1.** Timeline of COVID-19 vaccination policy, vaccine products, and eligibility criteria in Kosovo, March 2021–May 2023

| Date (Month/Year) | Vaccine Available | Vaccine Type(s) | Target Population / Eligibility |
| --- | --- | --- | --- |
| March 2021 | Primary vaccine series | ChAdOx1 nCoV-19 (Oxford–AstraZeneca) | Healthcare workers, adults ≥80 years |
| May 2021 | Primary vaccine series | Pfizer-BioNTech (BNT162b2) | Adults ≥65 years, chronic conditions |
| June 2021 | Primary vaccine series | AstraZeneca, Pfizer-BioNTech | All adults ≥18 years |
| September 2021 | First booster dose | Pfizer-BioNTech (BNT162b2) | Healthcare workers, elderly ≥65, immunocompromised |
| November 2021 | Primary vaccine series for 12–15-year-olds | Pfizer-BioNTech (BNT162b2) | Adolescents 12–15 years |
| December 2021 | First booster dose | Pfizer-BioNTech (BNT162b2) | Everyone ≥12 years who has received a primary vaccine series |
| July 2022 | Primary vaccine series and first booster dose | Pfizer-BioNTech (pediatric) | Children 5–11 years with parental consent |
| January 2023 | Additional booster doses | Pfizer-BioNTech XBB.1.5 (bivalent) | Everyone ≥12 years |

**Supplementary Table 2.** Adjusted Odds Ratios (aORs) corresponding to COVID-19 vaccine effectiveness estimates against hospitalization and severe outcomes in Kosovo, January 1, 2022–June 30, 2024

| **Days from last vaccine to symptom onset** | **Adjusted OR (95% CI)** | **Adjusted VE (95% CI)** |
| --- | --- | --- |
| *A) COVID VE against SARS-CoV-2-confirmed hospitalization for SARI among adults ≥ 18 years* | | |
| 14–179 | 0.28 [0.11–0.70] | 72% [30–89%] |
| 180–364 | 0.74 [0.41–1.33] | 26% [-33–59%] |
| < 365 | 0.55 [0.32–0.94] | 45% [6–68%] |
| *B) COVID-19 VE against SARS-CoV-2-confirmed hospitalization for SARI among adults ≥ 60 years* | | |
| 14–179 | 0.48 [0.18–1.31] | 52% [-31–82%] |
| 180–364 | 1.36 [0.64–2.90] | -36% [-190–36%] |
| < 365 | 0.95 [0.49–1.85] | 5% [-85–51%] |
| *C) COVID-19 VE against SARS-CoV-2-confirmed severe outcomes among adults ≥ 18 years* | | |
| 14–179 | 0.33 [0.09–1.14] | 67% [-14–91%] |
| 180–364 | 0.83 [0.33–2.11] | 17% [-111–67%] |
| < 365 | 0.58 [0.26–1.30] | 42% [-30–74%] |

**Supplementary Figure 1.** Annual COVID-19 vaccine effectiveness against SARS-CoV-2-confirmed hospitalization for SARI among adults A) ≥18 years old, B) ≥ 60 years old, and C) ≥18 years old with severe outcomes, by time since vaccination, COVID-19 vaccine effectiveness study in Kosovo, January 1, 2022–June 30, 2024
